# Supplementary material for: Epigenetic germline variants predict cancer prognosis and risk and distribute uniquely in topologically associating domains
Source: F1000Res. 2025 Jul 24;12:1083. Originally published 2023 Sep 1. [Version 2] doi: 10.12688/f1000research.139476.2 (PMC12826361; doi:10.12688/f1000research.139476.2)
Supplement: Peer review history

# Reviewer report: Reviewer response for version 2

**DOI:** 10.5256/f1000research.185383.r446700

**Published:** 2026-01-16

**Authors:** Kosuke Yamaguchi

**Affiliations:** 1 Molecular Cell Engineering Laboratory, National Institute of Genetics, Misima, Shizuoka, Japan

Methylation quantitative trait loci (meQTLs) are single nucleotide polymorphisms (SNPs) that are statistically associated with variation in DNA methylation levels at specific CpG sites. Such genetic-epigenetic interactions have attracted considerable interest in cancer research, as altered DNA methylation patterns can influence the expression of tumor suppressor genes and oncogenes. In addition, higher-order genome organization, including topologically associating domains (TADs), may further modulate the functional impact of genetic variants on epigenetic regulation in a context- and disease-specific manner.

 In this study, the authors integrate cancer-associated meQTLs with TAD organization. They report that transcriptionally active TADs tend to exhibit relative DNA hypomethylation, whereas inactive TADs are associated with DNA hypermethylation (Fig. 1). Furthermore, the authors observe that meQTLs are enriched at TAD boundaries, particularly at active-boundary-active, inactive-boundary-active, and active-boundary-inactive configurations (Fig. 2). The authors also report that oncogene-associated meQTLs are preferentially located within inactive TADs, whereas tumor suppressor gene-associated meQTLs are more frequently found in active TADs, based on randomization analyses (Fig. 3).

 The authors further identify 156 cancer gene-related clustered meQTLs (Table 1), among which 23 are reported to be significantly associated with cancer risk and survival across multiple cancer types in a pan-cancer model (Table 2). Breast cancer is highlighted as a representative example, supported by relatively large sample sizes from the UK Biobank and additional cohorts (Table 2, C50-C50). Using an XGBoost-trained model, the authors calculate a polygenic risk score (PRS) and suggest that this score may be associated with cancer risk (Fig. 4). The authors additionally attempt to isolate key contributing factors (MSH2, EZH2, and ASXL2) for cancer risk prediction; however, the expression levels of these genes do not show a significant correlation with PRS values (Fig. 5). Finally, the study proposes polygenic survival scores (PSS) derived from these meQTLs, suggesting that these scores may stratify cancer risk and survival beyond conventional clinical parameters (Fig. 6 and 7).

 Overall, this study provides conceptually interesting insights into the integration of genetic variation, DNA methylation, and three-dimensional genome organization in cancer risk assessment. However, several conclusions are not fully supported by the presented data, and important controls or additional analyses appear to be lacking. This reviewer therefore suggests that additional experiments and/or analyses would substantially strengthen the manuscript.

 Major Comments

 1. Figure and table labeling issues:

 Several figures and tables are not correctly labeled or lack essential information. The authors should carefully review all figures and tables to ensure clarity and completeness. Examples identified by this reviewer include:

- Figure 1A: The meaning of the X-axis label is unclear and should be explicitly defined.
- Table 2: No false discovery rate (FDR) or q-value information is provided; only p-values associated with ICD-10 codes are shown. If these p-values are intended to represent FDR-adjusted values, they should be clearly labeled as such.
- Table 2: The table legend describes beta values as correlation coefficients between meQTLs and promoter DNA methylation; however, it is not sufficiently clear from the table how these beta values should be interpreted in relation to the reported cancer risk and survival associations. Additional clarification would improve the readability of the table.
- Figure 5B: All Y-axis labels are shown as "MSH2 Expression (Z-score)," which appears to be incorrect.

2. DNA methylation source in Fig. 1B:

 In Fig. 1B, the authors analyze DNA methylation levels across 1,100 TADs shared among five cell lines (GM12878, HMEC, HUVEC, IMR90, and NHEK). As all these cell lines are derived from normal tissues, it is critical to clarify whether normal tissue DNA methylation data were used in this analysis. This reviewer requests explicit confirmation that normal (non-tumor) DNA methylation data from the TCGA database were used, rather than cancer-derived samples.

 3. Interpretation of Table 2 and biological linkage:

 Table 2 presents a list of meQTLs reported to significantly affect cancer risk and survival in a pan-cancer model. This reviewer assumes that the authors aim to establish a mechanistic link between meQTLs, TAD organization, promoter DNA methylation of cancer-related genes, gene expression changes, and cancer risk or survival. To strengthen this interpretation, this reviewer suggests:

- Adding gene expression comparisons between cancer and normal tissues for the genes listed in Table 2.
- Explicitly annotating whether each gene in Table 2 is classified as a tumor suppressor gene or an oncogene.

4. Evaluation of PRS specificity in Fig. 4:

 In Fig. 4, the authors show that a PRS derived from 23 TAD-associated meQTLs predicts breast cancer risk. However, meQTLs themselves have been reported as cancer-associated variants independent of TAD context. To specifically demonstrate the added value of TAD information, this reviewer recommends performing parallel PRS analyses using:

- Pan-cancer gene-related meQTLs, or
- All identified meQTLs,
- and directly comparing the prediction performance between TAD-associated meQTLs and these broader meQTL sets.

5. PSS comparison in Fig. 5:

 Similarly, for Fig. 5, the authors should calculate PSS values using pan-cancer gene-related meQTLs and compare their predictive performance with TAD-associated meQTL-based PSS. This comparison is necessary to demonstrate that incorporating TAD information provides added predictive value.

 6. Discussion: relevance of DNA methylation-CTCF interactions:

 Recent studies have reported that DNA methylation can directly affect CTCF binding, a key regulator of 3D genome organization. Incorporating this literature would strengthen the conceptual framework of the manuscript. The following references are suggested for discussion:

- PMID: 10839546
- PMID: 10839547
- PMID: 12461525
- PMID: 26257180
- PMID: 30948436
- PMID: 39180406

Minor Comments

 Introduction, second paragraph:

 "L1NE1" appears to be a typographical error and should be corrected to "LINE1."

 Figures 3B and 3C:

 The distinction between observed and random values is difficult to interpret. Using bar plots for both values with a clear legend would improve readability.

 Figure 4:

 The term polygenic risk score (PRS) is not defined prior to the use of the abbreviation. For readers outside the field, the full term should be introduced before abbreviation.

 Figure 4A:

 The X-axis labels "0" and "1" are not explained. It would be clearer to label these as "control" and "cancer," respectively.

 Text related to Fig. 5:

 Brief functional descriptions of MSH2 and EZH2 would improve clarity. MSH2 is a key component of DNA mismatch repair, and EZH2 is a core subunit of the PRC2 complex responsible for H3K27 methylation, both of which are closely linked to cancer biology.

 Page 9, Table 2 inconsistency:

 The manuscript states: "Out of the 155 SNPs, 21 passed the Benjamini-Hochberg adjusted FDR of less than 0.05 (Table 2)." However, Table 2 lists 23 SNPs. This discrepancy should be resolved. If an additional table exists, it should be included in the manuscript.

Is the work clearly and accurately presented and does it cite the current literature?

Partly

If applicable, is the statistical analysis and its interpretation appropriate?

Partly

Are all the source data underlying the results available to ensure full reproducibility?

Partly

Is the study design appropriate and is the work technically sound?

Partly

Are the conclusions drawn adequately supported by the results?

Partly

Are sufficient details of methods and analysis provided to allow replication by others?

Partly

Reviewer Expertise:

Epigenetic, Cell biology, Molecular cell biology.

I confirm that I have read this submission and believe that I have an appropriate level of expertise to confirm that it is of an acceptable scientific standard, however I have significant reservations, as outlined above.

# Reviewer report: Reviewer response for version 2

**DOI:** 10.5256/f1000research.185383.r432966

**Published:** 2026-01-07

**Authors:** Busra Unal

**Affiliations:** 1 Umraniye Training and Research Hospital, Istanbul, Turkey

The authors present an interesting and carefully conducted study. The manuscript is overall well written and provides valuable insights. However, several issues would benefit from clarification and further consideration

 1.Across the manuscript, the terms “epigenetic germline variants” and “cancer meQTLs” appear to be used somewhat interchangeably. It is not always clear if the authors are referring to germline variants with stable methylation effects across tissues and disease states, or to tumor-context–dependent meQTLs that may emerge within the altered epigenetic landscape of cancer. More consistent terminology would be helpful to clarify; which findings are reflecting underlying germline regulatory structure, and which findings are tumor-specific. This clarification is important because the manuscript’s conclusions regarding cancer risk inference may have potential clinical implications.

 2.The primary datasets used in this study (TCGA, UK Biobank, and DRIVE) are all substantially enriched for individuals of European ancestry. However, the manuscript does not evaluate if the reported findings generalize beyond these populations. Additionally, a discussion on how ancestry-related differences in linkage disequilibrium structure, baseline methylation landscapes, chromatin organization may influence both meQTL discovery and model performance is not fully addressed in the manuscript. Considering that epigenetic regulation and variant–methylation coupling can differ across populations, the absence of ancestry-stratified analyses or a conceptual consideration of these issues limits the generalisability of the work. I would recommend that the authors either incorporate ancestry-aware analyses or provide a discussion of how population structure may affect meQTL architecture and the broader applicability of their conclusions.

Is the work clearly and accurately presented and does it cite the current literature?

Yes

If applicable, is the statistical analysis and its interpretation appropriate?

I cannot comment. A qualified statistician is required.

Are all the source data underlying the results available to ensure full reproducibility?

Partly

Is the study design appropriate and is the work technically sound?

Yes

Are the conclusions drawn adequately supported by the results?

Yes

Are sufficient details of methods and analysis provided to allow replication by others?

Yes

Reviewer Expertise:

My area of research is hereditary cancer predisposition syndromes and disparities in genomic medicine application.

I confirm that I have read this submission and believe that I have an appropriate level of expertise to confirm that it is of an acceptable scientific standard.

# Reviewer report: Reviewer response for version 2

**DOI:** 10.5256/f1000research.185383.r400300

**Published:** 2025-08-12

**Authors:** Charu Mehta

**Affiliations:** 1 Basic Sciences Division, Fred Hutchinson Cancer Center, Seattle, Washington, USA

Thanks for your email. I have attached just a couple of minor comments. 

 In Fig 5A: meQTL associated with/near MSH2 has the highest “relative importance”. However, as the authors mention that MSH2 expression levels are not correlated with cancer risk in TCGA patients. Though the authors mention this in the discussion, it would be useful to mention the possibility of long range interactions wrt meQTL and target gene (e.g. through enhancer activity) here as well.

 Missing word on page 8: “predictive dirver meQTL was associated with MSH2…

Is the work clearly and accurately presented and does it cite the current literature?

Yes

If applicable, is the statistical analysis and its interpretation appropriate?

I cannot comment. A qualified statistician is required.

Are all the source data underlying the results available to ensure full reproducibility?

Partly

Is the study design appropriate and is the work technically sound?

Yes

Are the conclusions drawn adequately supported by the results?

Partly

Are sufficient details of methods and analysis provided to allow replication by others?

Yes

Reviewer Expertise:

NA

I confirm that I have read this submission and believe that I have an appropriate level of expertise to confirm that it is of an acceptable scientific standard.

# Reviewer report: Reviewer response for version 1

**DOI:** 10.5256/f1000research.152752.r233315

**Published:** 2024-02-08

**Authors:** Chiara Herzog

**Affiliations:** 1 Universitat Innsbruck, Innsbruck, Tyrol, Austria

The study by Goudarzi et al. provides interesting and new insights into the association of meQTLs and TAD regions the genome, and investigates the capacity of meQTLs to predict cancer status and survival.

 Overall the study is well done and clearly presented but I have a few comments and suggestions for improvement.

 Major:

 - In the introduction and discussion the authors state “This study investigated the relationship between epigenetic factors like chromatin structure and DNA methylation and genetic variation in the context of cancer”. While the authors indeed investigate methylation and TADs in the first part of the manuscript, the majority of the predictors focus on genetic loci and not epigenetics or their interaction. Arguably, one of the most interesting aspects of meQTLs in their capacity for risk prediction are their modulated methylation levels and potential to reflect the integration of genetic and dynamic nonheritable factors (such as due to aging or lifestyle factors), but this was not looked at in detail. Could the authors comment on how meQTLs might be modulated by nonheritable factors as well as genetic factors, and e.g. look into methylation at these sites in cancers or samples preceding cancer?

 - Along these lines, it might in the future be interesting to develop dynamic cancer risk predictors as opposed to static tools (such as the PRSs), which might be enabled by nongenetic ‘omics’. Could the authors discuss the potential of these and how their findings might contribute to this (i.e. how meQTLs might contribute to dynamic risk monitoring)?

 Minor:

 - The authors describe a PCA but do not show any figures or supporting data. Could the authors either add a statement that no data are shown in the text, or (preferred) provide these data in the supplementary information?

 - Previous breast cancer PRSs (not based on meQTL) such as the PRS313 have already shown that they may be biased towards certain subtypes - it might be worth mentioning these prior models (e.g. Mavaddat et al 2019) when discussing the current study’s findings in context.

 - What was the ROC AUC (and 95% CI) of the cancer risk score (Figure 4A)?

 - Can the authors explain the discrepancy of the more ‘linear’ increase in risk in the UKBB compared to DRIVE (Figure 4b versus 4c)?

 - Figure 6/TCGA survival: It might also be interesting to look at recurrence-free survival in addition to overall survival.

 - In the section Oncogenes and tumor suppressor gene-related (…): capitalise **L** in ‘Clumped cancer mQT **l**s’

 - Figures:

     - Figure 1b: Could the authors also indicate in the Figure legend that this is a Kruskal-Wallis p value.

     - Figure 1c - For interpretability, it might be helpful to add at least y axis grid lines behind box plots. The effects may be significant and is visualised with the violin density plot, but is difficult to see using box plot.

     - Figure 2 - takes some time to understand upon first reading. It might be helpful to label the blue bars in B and C with a legend ‘expected’ and the red dot as ‘observed’ to make it easier to grasp quickly.

     - Figure 4: The text in the caption for A refers to a ‘Figure 8’ that does not exist. Please check this.

Is the work clearly and accurately presented and does it cite the current literature?

Yes

If applicable, is the statistical analysis and its interpretation appropriate?

Yes

Are all the source data underlying the results available to ensure full reproducibility?

Yes

Is the study design appropriate and is the work technically sound?

Yes

Are the conclusions drawn adequately supported by the results?

Partly

Are sufficient details of methods and analysis provided to allow replication by others?

Yes

Reviewer Expertise:

epigenetics and cancer risk prediction

I confirm that I have read this submission and believe that I have an appropriate level of expertise to confirm that it is of an acceptable scientific standard, however I have significant reservations, as outlined above.

# Response

**Published:** 2025-07-20

**Authors:** Hannah Carter

**Affiliations:** University of California, San Diego, USA

The study by Goudarzi et al. provides interesting and new insights into the association of meQTLs and TAD regions of the genome, and investigates the capacity of meQTLs to predict cancer status and survival.

 Overall the study is well done and clearly presented but I have a few comments and suggestions for improvement.

 Major:

 - In the introduction and discussion the authors state “This study investigated the relationship between epigenetic factors like chromatin structure and DNA methylation and genetic variation in the context of cancer”. While the authors indeed investigate methylation and TADs in the first part of the manuscript, the majority of the predictors focus on genetic loci and not epigenetics or their interaction. Arguably, one of the most interesting aspects of meQTLs in their capacity for risk prediction are their modulated methylation levels and potential to reflect the integration of genetic and dynamic non heritable factors (such as due to aging or lifestyle factors), but this was not looked at in detail. Could the authors comment on how meQTLs might be modulated by non heritable factors as well as genetic factors, and e.g. look into methylation at these sites in cancers or samples preceding cancer?

*We agree with the reviewer that it is important to consider potential for interaction with non-heritable factors. We have modified our discussion to acknowledge the need to evaluate non-heritable factors as follows:*

*“There are also a number of non-genetic risk factors that act by modifying DNA methylation levels and which could interact with genetic regulation. These include aging, exercise, stress, diet and obesity, and a broad variety of environmental exposures. In our analysis, age had the highest impact on DNA methylation modulation, however, as age and sex were the only clinical factors for the majority of our study, future analysis of other non-genetic factors in relation to genetic regulators of DNA methylation are merited.”*

 - Along these lines, it might in the future be interesting to develop dynamic cancer risk predictors as opposed to static tools (such as the PRSs), which might be enabled by nongenetic ‘omics’. Could the authors discuss the potential of these and how their findings might contribute to this (i.e. how meQTLs might contribute to dynamic risk monitoring)?

*This is a nice suggestion. We have added the following to the discussion:*

*“Future efforts could integrate dynamic methylation changes due to these non-genetic factors with static polygenic scores such as we describe here to provide a more accurate estimate of risk. This type of approach could benefit in particular from non-invasive biomarkers, such as cell free DNA methylation from blood, though studies will be needed to establish the cumulative effect of dynamic exposures and the extent to which they can be accurately evaluated from cell free DNA.”*

*Ref:*

*Yousefi, P.D., Suderman, M., Langdon, R. et al. DNA methylation-based predictors of health: applications and statistical considerations. Nat Rev Genet 23, 369–383 (2022). https://doi.org/10.1038/s41576-022-00465-w*

 Minor:

 - The authors describe a PCA but do not show any figures or supporting data. Could the authors either add a statement that no data are shown in the text, or (preferred) provide these data in the supplementary information?

*We have added the PCA figure as Supplementary Figure 1.*

 - Previous breast cancer PRSs (not based on meQTL) such as the PRS313 have already shown that they may be biased towards certain subtypes - it might be worth mentioning these prior models (e.g. Mavaddat et al 2019) when discussing the current study’s findings in context.

*We have now included the following text:*

*“Indeed, classic polygenic risk scores for breast cancer have shown bias for predicting certain subtypes (Mavaddat et al). Lakeman et al 2020 demonstrated that women in the highest 1% of risk showed a 4.37-fold increased risk for ER-positive disease but only a 2.78-fold increased risk for ER-negative disease compared to the middle quintile showing bias in certain subtypes.”*

*Refs*

*Mavaddat N, Michailidou K, Dennis J, Lush M, Fachal L, Lee A, Tyrer JP, Chen TH, Wang Q, Bolla MK, Yang X. Polygenic risk scores for prediction of breast cancer and breast cancer subtypes. The American Journal of Human Genetics. 2019 Jan 3;104(1):21-34.*

*Lakeman, I.M.M., Rodríguez-Girondo, M., Lee, A. et al. Validation of the BOADICEA model and a 313-variant polygenic risk score for breast cancer risk prediction in a Dutch prospective cohort. Genet Med 22, 1803–1811 (2020). https://doi.org/10.1038/s41436-020-0884-4*

 - What was the ROC AUC (and 95% CI) of the cancer risk score (Figure 4A)? 

*THE DRIVE AUC 0.5534 with 95% CI between [0.5505, 0.5563]. This has now been added to the manuscript. We note that this PRS is based solely upon meQTLs near driver genes and is not expected to be a strong predictor of risk relative to more comprehensive breast cancer polygenic scores. Rather we sought to reproduce effects on risk attributable solely to driver meQTLs in an independent cohort.*

 - Can the authors explain the discrepancy of the more ‘linear’ increase in risk in the UKBB compared to DRIVE (Figure 4b versus 4c)?

*This discrepancy could be due to inherent differences in the composition of the two datasets. UKBB comes predominantly from volunteers in the UK and is more representative of disease incidence in the general population. The DRIVE study was designed specifically to analyze breast cancer risk, and therefore individuals were included to either represent breast cancer or serve as non-breast cancer controls.Breast cancer status in the UKBB was defined based on ICD-10 codes, and we excluded individuals from the controls if they had any ICD10 code associated with neoplasms, but the number of cases relative to controls was unbalanced for UKBB whereas it was balanced for DRIVE.  The difference in linearities between the patients could be due to differences in diversity in genotype and phenotype in the UKBB cohort compared to the DRIVE cohort.  There could also be discrepancies in environmental risk factors between these cohorts.*

 - Figure 6/TCGA survival: It might also be interesting to look at recurrence-free survival in addition to overall survival.

*We now include an analysis of disease free intervals as Figure SX. The methods have been updated accordingly.*

 - In the section Oncogenes and tumor suppressor gene-related (…): capitalise L in ‘Clumped cancer mQTls’

*We have now corrected this.* 

 - Figures:

 - Figure 1b: Could the authors also indicate in the Figure legend that this is a Kruskal-Wallis p value.

*We have updated the figure legend to reflect the test used.*

 - Figure 1c - For interpretability, it might be helpful to add at least y axis grid lines behind box plots. The effects may be significant and is visualised with the violin density plot, but are difficult to see using box plots.

*We added gridlines as suggested.*

 - Figure 2 - takes some time to understand upon first reading. It might be helpful to label the blue bars in B and C with a legend ‘expected’ and the red dot as ‘observed’ to make it easier to grasp quickly.

*We have updated the legends to read observed and expected.*

 - Figure 4: The text in the caption for A refers to a ‘Figure 8’ that does not exist. Please check this.

*Thank you for catching this. The erroneous figure reference has been removed from the caption.*

# Reviewer report: Reviewer response for version 1

**DOI:** 10.5256/f1000research.152752.r233320

**Published:** 2024-01-17

**Authors:** Charu Mehta

**Affiliations:** 1 Basic Sciences Division, Fred Hutchinson Cancer Center, Seattle, Washington, USA

"To obtain independent meQTLs, we clumped related meQTLs based on linkage disequilibrium using PLINK.

 Out of the 1.2 million SNPs, 60,602 remained after LD pruning (Table 1)."

Comment: Cite the database used as a source of meQTLs? In the discussion, authors cited a database (46) but it is unclear if this is the same database they used to identify meQTLs.

 "(A) 5 state-based K-Means clustering of common TAD domains (n=1100) between 5 human cell lines (GM12878,

 HMEC, HUVEC, IMR90, and NHEK). Purple indicates TADs classified as a “Mixed”, Gray as “Inactive-1”, Light Blue as

 “Active-1”, Orange as “Active-2”, and Red as “Inactive-2”. Combining active and inactive categories leads to 222 Active,

 626 Inactive, and 252 Mixed TADs."

 Comment: what are the x- and y-axes labels in 1A? Where are the five cell types indicated?

 Comment: What does ‘other’ mean in Table 1?

 "Distributions suggested an increase in density of clumped meQTLs when transitioning from active

 to inactive regions, and conversely, a decrease from inactive to active regions (Kruskal-Wallis ANOVA, p-value<0.05)

 when compared to the randomly shuffled distribution, but no shift in density for Active-Boundary-Active and Inactive-

 Boundary-Inactive categories (Figure 2B-D)."

 Comment: So what does any of this suggest??? expand?

 "In total, 103 oncogenes and 223 TSGs were used for this analysis, where only 67 of them contained meQTL-affecting CpG probes in their promoter regions (i.e. 49 TSGs and 18 oncogenes)."

 Comment: This suggests CpGs affect meQTLs but it’s the other way round.

 "Clumped cancer meQTls were further narrowed to those associated with the methylation status of CpG probes located within the promoter regions of cancer driver genes including oncogenes and tumor suppressor genes (TSGs) from the

 COSMIC database.” Comment: Also clarify if the correlation between methylation status of CpG vs meQTLs is also observed in normal tissues or only cancer tissues?

 "Overall, cancer meQTLs near 29 cancer genes were included in the model. The most predictive driver meQTL was associated MSH2, a gene associated with Lynch syndrome and increased risk of breast cancer.

 Polymorphic variation affecting the expression of EZH2, the second most informative feature, has also been linked to breast cancer risk.

 ASXL2 may be required for estrogen receptor alpha (ERa) activation in ERa positive breast cancers. Notably, EZH2 overexpression has been linked more strongly to triple negative breast suggesting that the model includes features predictive of multiple subtypes.”

 Comment: 1. Since some of these meQTLs lie close to genes involved in epigenetic modifications --- have you looked if these are in the enhancer or otherwise defined regulatory domains?

 2. Are these genes (MSH2, EZH2, ASXL2) known to be upregulated or downregulated in these risk cases? Does that agree with the prediction according to meQTLs?

Is the work clearly and accurately presented and does it cite the current literature?

Yes

If applicable, is the statistical analysis and its interpretation appropriate?

I cannot comment. A qualified statistician is required.

Are all the source data underlying the results available to ensure full reproducibility?

Partly

Is the study design appropriate and is the work technically sound?

Yes

Are the conclusions drawn adequately supported by the results?

Partly

Are sufficient details of methods and analysis provided to allow replication by others?

Yes

Reviewer Expertise:

My area of research is gene regulation. I am able to assess significant parts of this manuscript, however, I am not a statistician or computational biologist, so I cannot speak to the soundness of their methods.

I confirm that I have read this submission and believe that I have an appropriate level of expertise to confirm that it is of an acceptable scientific standard, however I have significant reservations, as outlined above.

# Response

**Published:** 2025-07-20

**Authors:** Hannah Carter

**Affiliations:** University of California, San Diego, USA

"To obtain independent meQTLs, we clumped related meQTLs based on linkage disequilibrium using PLINK. Out of the 1.2 million SNPs, 60,602 remained after LD pruning (Table 1)."

 Comment: Cite the database used as a source of meQTLs? In the discussion, authors cited a database (46) but it is unclear if this is the same database they used to identify meQTLs.

*The data used as a source of the meQTLs are the Pancan-meQTLs from the cited number 46 [Gong J, et al.: Pancan-meQTL: a database to systematically evaluate the effects of genetic variants on methylation in human cancer. Nucleic Acids Res. 2019; 47: D1066–D1072]*

*We have modified the text to clarify this as follows:*

*“To obtain independent meQTLs, we clumped related meQTLs from Gong et al (46) based on linkage disequilibrium using PLINK. Out of the 1.2 million SNPs, 60,602 remained after LD pruning (Table 1).”*

 "(A) 5 state-based K-Means clustering of common TAD domains (n=1100) between 5 human cell lines (GM12878, HMEC, HUVEC, IMR90, and NHEK). Purple indicates TADs classified as a “Mixed”, Gray as “Inactive-1”, Light Blue as “Active-1”, Orange as “Active-2”, and Red as “Inactive-2”. Combining active and inactive categories leads to 222 Active, 626 Inactive, and 252 Mixed TADs."

 Comment: what are the x- and y-axes labels in 1A? Where are the five cell types indicated?

 The x-axis are the 15 chromatin states that are used to cluster the TADs. The y-axis are all 1100 TADs that were used for the analysis and the 5 cell types are indicated through the legend shown on the left. The different colors represent the “Mixed”, “Inactive-1”, “Active-1”, “Active-2”, “Inactive-2”.

*We have updated the figure legend as follows:*

*“(A) 5 state-based K-Means clustering of common TAD domains shared across 5 human cell lines (GM12878, HMEC, HUVEC, IMR90, and NHEK). Shared TAD domains are on the y-axis (n=1100) and are grouped according to 15 chromatin states (x-axis). K-means clusters are shown as a side bar along the y-axis. Purple indicates TADs classified as a “Mixed”, Gray as “Inactive-1”, Light Blue as “Active-1”, Orange as “Active-2”, and Red as “Inactive-2”. Combining active and inactive categories leads to 222 Active, 626 Inactive, and 252 Mixed TADs.”*

 Comment: What does ‘other’ mean in Table 1?

*Other represents all meQTLs that are in the inter-TAD region that aren’t technically in the boundary region, since we defined the boundary region as +/- 50kb around the TAD boundaries. We have added a note to the table legend to clarify this.*

*“Other indicates meQTLs that are in the inter-TAD region but do not fall within the boundary region as defined.”*

 "Distributions suggested an increase in density of clumped meQTLs when transitioning from active to inactive regions, and conversely, a decrease from inactive to active regions (Kruskal-Wallis ANOVA, p-value<0.05) when compared to the randomly shuffled distribution, but no shift in density for Active-Boundary-Active and Inactive-Boundary-Inactive categories (Figure 2B-D)."

 Comment: So what does any of this suggest??? expand?

*We have tried to further expand our interpretation of this observation in the discussion as follows:*

*“It is of note that TAD boundaries conserved across cell types are reportedly highly enriched for evolutionary constraint and complex trait heritability.10 Our data suggest that variability in gene expression due to meQTLs is also evolutionarily more constrained in and around active TADs and their boundaries, consistent with these TAD boundaries playing a critical role in development (47). These results may suggest that TAD boundaries play a role in making the recruitment of regulatory machinery more specific, particularly as it pertains to DNA methylation.”*

 "In total, 103 oncogenes and 223 TSGs were used for this analysis, where only 67 of them contained meQTL-affecting CpG probes in their promoter regions (i.e. 49 TSGs and 18 oncogenes)."

 Comment: This suggests CpGs affect meQTLs but it’s the other way round.

*We changed “meQTL-affecting” to “meQTL-associated”.*

 "Clumped cancer meQTls were further narrowed to those associated with the methylation status of CpG probes located within the promoter regions of cancer driver genes including oncogenes and tumor suppressor genes (TSGs) from the COSMIC database.” 

 Comment: Also clarify if the correlation between methylation status of CpG vs meQTLs is also observed in normal tissues or only cancer tissues?

*The meQTLs generated by Gong et al were based on methylation status at CpG markers as measured in bulk tumor tissues which generally include a mixture of tumor and normal cells (stroma and immune infiltrates). Nonetheless, it is not clear whether the effects detected here are biased toward effects on methylation that are positively selected in tumors which might not be reflected in normal tissues. This limitation is described in the discussion:*

*“First, the meQTLs utilized for this study are derived from a study of tumors 46 which could be biased toward detecting meQTLs associated with DNA methylation events that are positively selected in tumors. “*

*and*

*“In future studies, it would be of interest to study meQTL trends in normal tissue samples to see if enrichment patterns associated with cancer genes are driven by selection in tumors, or highlight evolutionary constraints more broadly associated with human health that coincidentally are advantageous for tumor development.”*

*We also believe that our original phrasing was confusing here. We have rephrased as follows:*

*"Clumped cancer meQTLs were further narrowed to those whose corresponding affected CpG probes were within the promoter regions of cancer driver genes including oncogenes and tumor suppressor genes (TSGs) from the COSMIC database.”*

 "Overall, cancer meQTLs near 29 cancer genes were included in the model. The most predictive driver meQTL was associated MSH2, a gene associated with Lynch syndrome and increased risk of breast cancer.

 Polymorphic variation affecting the expression of EZH2, the second most informative feature, has also been linked to breast cancer risk. ASXL2 may be required for estrogen receptor alpha (ERa) activation in ERa positive breast cancers. Notably, EZH2 overexpression has been linked more strongly to triple negative breast suggesting that the model includes features predictive of multiple subtypes.”

 Comment:

 1. Since some of these meQTLs lie close to genes involved in epigenetic modifications --- have you looked if these are in the enhancer or otherwise defined regulatory domains?

*The challenge here is that we do not know if the tag meQTL SNP is actually the causal SNP. We focused on meQTLs that affect CpG probes within the promoter regions of cancer genes but that does not preclude the possibility that they are affecting an enhancer. We have added this as a limitation of our study:*

*“For risk prediction, we focused on meQTLs and their corresponding CpG probes that are overlapping the promoter regions of known cancer genes, however we cannot be sure that these meQTLs are not also affecting other genes in the region, for example through effects on enhancer activity. ”*

 2. Are these genes (MSH2, EZH2, ASXL2) known to be upregulated or downregulated in these risk cases?

 Does that agree with the prediction according to meQTLs?

*Establishing the mechanism by which meQTLs drive risk would require tissue-relevant gene expression, methylation measurements and genotypes in a group of individuals prior to them developing breast cancer. In established breast cancers in TCGA, the relationship of gene expression with methylation is confounded by somatic alterations and copy number events and general dysregulation of gene expression networks making it difficult to determine what proportion of gene expression is attributable to meQTLs. We have added a statement about the need to further investigate meQTL effects on oncogene and tumor suppressor gene expression in healthy breast tissue to establish how these effects relate to cancer risk.*

*“More direct mechanistic insight might be gained by studying expression, genotype and methylation in healthy and pre-cancerous breast tissues and cell types. Studying the average expression of MSH2, EZH2, and ASXL2 within TCGA patients stratified by meQTL risk PRS suggested a potential decrease in expression of ASXL2 and EZH2 from in the highest PRS quantile relative to the lowest while MSH2 did not show much difference (Figure 5B). However, this difference needs to be studied further with more specific tumor sub-type stratification and cell type-specific expression. ”*

*Figure 5 and caption have been updated as follows:*

*“A) Features are ranked according to their contribution to classifier predictive performance. Total importances sum to 1. B) Average expression of ASXL2, EZH2 and MSH2 in TCGA breast cancer samples, stratified by PRS quantile.”*
